# *Aedes aegypti* bred from vegetables collected in localities exposed to pyriproxyfen in Sullana, Peru, 2024

**DOI:** 10.17843/rpmesp.2024.414.14220

**Published:** 2024-10-22

**Authors:** Archi Alejandro Ruiz-Polo, Rosa Elena Santillan-Valdivia, Jayder Martín Temoche-Villarreyes, Lya Emilia Niño-Mendoza, Jesús Geraldine Peña de la Cruz

**Affiliations:** 1 Center for Research and Training in Entomology, Luciano Castillo Colonna Sub Regional Health Directorate. Sullana, Piura, Peru Center for Research and Training in Entomology Luciano Castillo Colonna Sub Regional Health Directorate Sullana Piura Perú; 2 Vector Surveillance and Control Program, Luciano Castillo Colonna Sub Regional Health Directorate, Sullana, Piura, Peru. Vector Surveillance and Control Program Luciano Castillo Colonna Sub Regional Health Directorate Sullana Piura Perú

To the editor. Pyriproxyfen is a chemical compound that, although it is not a hormone, biologically behaves as a regulator of insect growth, preventing the final transformation of the pre-adult (pupa) to adult stages; therefore, it has been widely used in focal control and/or control of *A. aegypti* breeding sites in Peru. In this context, it has recently been reported that *A. aegypti* populations in the north of our country are still susceptible to this chemical compound ^1^. However, these results are often obtained from *in vitro* bioassays ^2^, which use strains of up to two generations obtained from eggs collected and preserved for several months. This is a very important factor if we considered that, genetic variations appear during epidemic scenarios, which give rise to the formation of subpopulations and subspecies with biological changes in the levels of anthropophilia, resistance to chemical controllers and vectorial competition ^3^^,^^4^. In addition, studies on the effect and residuality of pyriproxyfen on culicidae show a contrast between *in vitro* and *in situ* results, since the percentages of inhibition of adult emergence are close to 100%using doses of 0.01 and 0.02 ppm. However, under field conditions and with the same doses, the inhibition percentage is lower ^5^^,^^6^. Nevertheless, the surviving adults present malformations that cause their eventual death ^7^. In this regard, we present this scientific letter in which we determine the adult morphology of *A. aegypti* bred from larvae collected in localities exposed to pyriproxyfen in Sullana, Peru.

We conducted an *in vitro,* descriptive research with a quantitative approach and non-experimental design, in the insectary of the Research and Training Center in Entomology - CICE of the Subregional Health Directorate Luciano Castillo Colonna (DSRSLCC) of Sullana of the regional government of Piura.

We analyzed 1918 larvae between L3 and L4 stages collected during June 2024 in different localities of the province of Sullana (Santa Teresita, El Obrero and Bellavista) that have been exposed to pyriproxyfen 0.5% (LIMITOR GR) through hatchery control and/or focal control (FC) between January and June 2024. The larvae were provided by the DSRSLCC surveillance and vector control program, who have been performing the control at doses of 0.01 ppm (0.01 mg/L) of active ingredient (AI). The controls were carried out on the following dates: In Santa Teresita on 11/01 (CF-1), 02/03 (CF-2) and 07/05 (CF-3). In El Obrero on 01/29 (CF-1), 02/13 (CF-2) and 05/03 (CF-3). In Bellavista on 10/02 (CF-1), 13/04 (CF-2) and 19/06 - 05/07 (CF-3).

*Aedes aegypti* were raised from larvae to adults in 26 days. We used thirteen plastic trays with 500 mL of pipe water rested for 24 hours for the larvae, we placed 150 larvae in twelve trays each and 118 in another tray. The complementary abiotic factors were 0.2 mg/L of residual chlorine in water and an environmental microclimate with 26.2±17 °C temperature and 88.6±19 % relative humidity. The first factor was measured with a DR900 colorimeter (0.01 m/L error) and the second with a Taylor Light 1523 thermohygrometer (water temperature was not evaluated). Water in the trays was changed daily and after each change we dispensed powdered feed (mixture of chicken purine with commercial brewer’s yeast sieved at a No. 60 of 250 uM). Feeding stopped upon reaching the pupal stage, and the pupae were transferred to polyethylene cups containing 200 milliliters of water. Then, using stylet, tweezers and the Pan American Health Organization’s manual of ecology and biology of *A. aegypti*^8^, pupae and emerging adults were phenotypically analyzed for leg shape (posterior, middle and anterior), proboscis, antennae, abdomen, cephalothorax, head and maxillary palps.

Morphological data were photoregistered and analyzed using a ZEISS Stemi DV4 stereoscope coupled to the camera of an Honor X7 model CMA-LX3 smartphone. Quantitative data were recorded and analyzed in Excel spreadsheets version 2021.

We found that the larvae reached the pre-adult (pupa) and adult stages of the biological cycle of *A. aegypti*. However, both stages presented mortality. The 1918 (100%) analyzed larvae reached L4 stage; however, 793 (41.4%) died and only 1125 (58.6%) reached the pupal stage. Likewise, of the total number of pupae, 774 (68.8%) died and 351 (31.2%) emerged as adults. Finally, of the total number of adults, 214 (60.9%) showed malformations and 137 (39.1%) did not. It is important to note that the number of adults without malformations is very relevant (39.1%), considering that pyriproxyfen acts by inhibiting the changes of stages from larva to pupa and adult, that is, by interrupting the biological cycle of *A. aegypti*, and it also has a residual effect.

**Figure 1:**
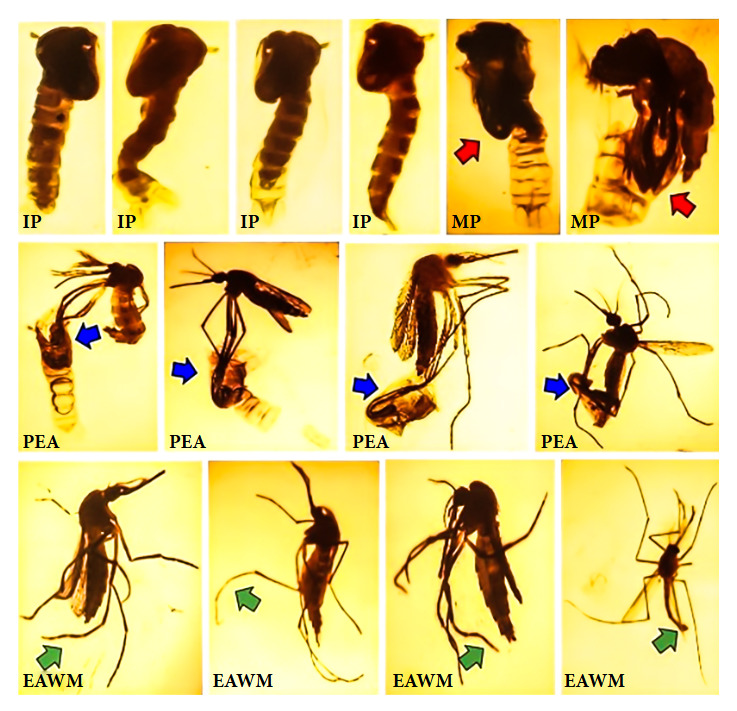
Pupae and dead adults from areas exposed to pyriproxyfen in Sullana, Peru. IP. Immature pupa. MP. Mature pupa (red arrow points to legs, abdomen and/or thorax in emergence). PEA. Partially emerged adult (blue arrow points to tarsi of posterior, middle and anterior legs within pupal exuvia). EAWM. Emerged adult with malformations (green arrow points to atrophied abdomen and bent tarsi of posterior, middle and anterior legs).

Figure 1 shows specimens with pupal and adult stage malformations.

This study has the technological limitation of high-resolution digital stereoscopes which are used to observe defined morphological characteristics. However, it was possible to find and differentiate malformations.

In conclusion, the larvae collected during the month of June in localities exposed to pyriproxyfen from January to June 2024, reached the pupal and adult stages. We found that 137 (7.1%) adult specimens did not present malformations, inferring the existence of resistant subpopulations that need to be studied extensively. This is a very relevant finding considering that pyriproxyfen is a chemical agent with residual effect. This information contributes as a precedent to future research and to the development of efficient and effective strategies for focal control and/or control of breeding sites.
