# Supplementary material for: Aedes aegypti bred from vegetables collected in localities exposed to pyriproxyfen in Sullana, Peru, 2024
Source: Rev Peru Med Exp Salud Publica. 2024 Oct 22;41(4):442–4. doi: 10.17843/rpmesp.2024.414.14220 (PMC11797588; doi:10.17843/rpmesp.2024.414.14220)
Supplement: Translation

# Translation: Morfología adulta de *Aedes aegypti* criados desde larvas recolectadas en localidades expuestas a pyriproxyfen en Sullana, Perú, 2024

**Authors:** Archi Alejandro Ruiz-Polo, Rosa Elena Santillan-Valdivia, Jayder Martín Temoche-Villarreyes, Lya Emilia Niño-Mendoza, Jesús Geraldine Peña de la Cruz

**Affiliations:** 1 Centro de Investigación y Capacitación en Entomología, Dirección Sub Regional de Salud Luciano Castillo Colonna. Sullana, Piura, Perú.; 2 Programa de Vigilancia y Control Vectorial, Dirección Sub Regional de Salud Luciano Castillo Colonna, Sullana, Piura, Perú.

Sr. Editor. El pyriproxyfen es un compuesto químico que, si bien no es una hormona, biológicamente se comporta como un regulador en el crecimiento de insectos, impidiendo la transformación final de los estadios preadulto (pupa) a adulto; por lo que viene siendo ampliamente usado en el control focal y/o control de criaderos de *A. aegypti* en el Perú. En ese contexto, recientemente se ha informado que poblaciones de *A. aegypti* del norte de nuestro país aún son susceptibles a este compuesto químico ^1^. Sin embargo, estos resultados muchas veces se obtienen de bioensayos *in vitro*^2^, en los que se emplean cepas de hasta dos generaciones obtenidas a partir de huevos recolectados y conservados por varios meses. Un factor muy relevante si se considera qué, durante escenarios epidémicos se manifiestan variaciones genéticas que dan lugar a la formación de subpoblaciones y subespecies con cambios biológicos en los niveles de antropofilia, resistencia a controladores químicos y competencia vectorial ^3^^,^^4^. Además, estudios sobre el efecto y residualidad del pyriproxyfen en culícidos señalan una contraposición entre resultados *in vitro* e *in situ*, ya que usando dosis de 0,01 y 0,02 ppm en laboratorio, los porcentajes de inhibición de la emergencia del adulto son cercanos al 100%. Sin embargo, en condiciones de campo y con las mismas dosis, el porcentaje de inhibición es menor ^5^^,^^6^. No obstante, los adultos sobrevivientes presentan malformaciones que causan su eventual muerte ^7^. En ese sentido, presentamos esta carta científica en la que determinamos la morfología adulta de *A. aegypti* criados desde larvas recolectadas en localidades expuestas a pyriproxyfen en Sullana, Perú.

Realizamos una investigación *in vitro* con nivel descriptivo, enfoque cuantitativo y diseño no experimental, que llevamos a cabo en el insectario del Centro de Investigación y Capacitación en Entomología - CICE de la Dirección Subregional de Salud Luciano Castillo Colonna (DSRSLCC) de Sullana del gobierno regional de Piura.

Analizamos 1918 larvas entre los estadios L3 y L4 recolectadas durante el mes de junio del año 2024 en distintas localidades de la provincia de Sullana (Santa Teresita, El Obrero y Bellavista) que se han encontrado expuestas a pyriproxyfen 0,5 % (LIMITOR GR) mediante el control de criaderos y/o control focal (CF) entre enero y junio del año 2024. Las larvas han sido proporcionadas por el programa de vigilancia y control vectorial de la DSRSLCC, quienes han venido realizando el control en dosis de 0,01 ppm (0,01 mg/L) de ingrediente activo (IA). Los controles se realizaron en las siguientes fechas: En Santa Teresita el 11/01 (CF-1), 02/03 (CF-2) y 07/05 (CF-3). En El Obrero el 29/01 (CF-1), 13/02 (CF-2) y 03/05 (CF-3). En Bellavista el 10/02 (CF-1), 13/04 (CF-2) y 19/06 - 05/07 (CF-3).

La crianza de *Aedes aegypti* desde larvas ha adultos la realizamos en 26 días. Para las larvas empleamos trece bandejas de plástico con 500 mL de agua de tubería reposada por 24 horas, en doce bandejas colocamos 150 larvas por cada una y en otra bandeja 118. Los factores abióticos complementarios fueron un cloro residual de 0,2 mg/L en agua y un microclima ambiental con 26.2±17 ºC de temperatura y 88.6±19 % de humedad relativa. El primer factor se midió con un colorímetro DR900 (error de 0,01 m/L) y el segundo con un termohigrómetro Taylor Light 1523 (la temperatura del agua no se evaluó). El agua de las bandejas se cambió diariamente y después de cada cambio dispensamos alimento en polvo (mezcla de purina de gallina con levadura de cerveza comercial tamizada en una N.º 60 de 250 uM). Al llegar al estadio de pupa, se omitió la alimentación y las pupas se trasladaron a vasos de polietileno con 200 mililitros de agua. Luego, empleando estiletes, pinzas y el manual de ecología y biología de *A. aegypti* de la Organización Panamericana de la Salud ^8^, pupas y adultos emergentes fueron analizados fenotípicamente en la forma de las patas (posteriores, medias y anteriores), probóscide, antenas, abdomen, cefalotórax, cabeza y palpos maxilares.

Los datos morfológicos los fotoregistramos y analizamos mediante un estereoscopio modelo Stemi DV4 marca ZEISS acoplado a la cámara de un Smartphone Honor X7 modelo CMA-LX3. Los datos cuantitativos los registramos y analizamos en hojas de cálculo del Excel versión 2021.

En nuestros resultados, observamos que las larvas analizadas alcanzaron los estadios de pre-adulto (pupa) y adulto que componen el ciclo biológico de *A. aegypti.* No obstante, ambos estadios presentaron mortalidad. Las 1918 (100%) larvas analizadas alcanzaron el estadio L4, sin embargo, 793 (41,4%) murieron y solo 1125 (58,6%) llegaron al estadio de pupa. Así mismo, del total de pupas, 774 (68,8 %) murieron y 351 (31,2%) emergieron en adultos. Finalmente, del total de adultos, 214 (60,9%) presentaron malformaciones y 137 (39,1%) no las presentaron. Es importante resaltar que el número de adultos sin malformaciones observado es muy relevante (39,1%) considerando que el pyriproxyfen actúa inhibiendo los cambios de estadios desde larva a pupa y adulto, es decir interrumpiendo el ciclo biológico de *A. aegypti*, y que además posee efecto residual.

En la figura 1 se muestran especímenes con malformaciones en estadio de pupa y adulto.

**Figura 1:** Pupas y adultos muertos de zonas expuestas a pyriproxyfen en Sullana, Perú. PI. Pupa inmadura. PM. Pupa madura (flecha roja señala patas, abdomen y/o tórax en emergencia). AEP. Adulto emergido parcialmente (flecha azul señala tarsos de patas posteriores, medias y anteriores dentro de exuvia pupal). AECM. Adulto emergido con malformaciones (flecha verde señala abdomen atrofiado y tarsos doblados de las patas posteriores, medias y anteriores).

El presente estudio posee la limitación tecnológica de estereoscopios digitales de alta resolución con los que pueda observar características morfológicas definidas. Sin embargo, sí se logró observar y diferenciar malformaciones.

En conclusión, las larvas que se recolectaron durante el mes de junio en localidades expuestas a pyriproxyfen desde enero a junio del 2024, alcanzaron el estadio de pupa y adulto, siendo este último donde 137 (7,1%) especímenes no presentaron malformaciones, infiriendo la existencia de subpoblaciones resistentes que necesitan ser ampliamente estudiadas. Un hallazgo muy relevante si se considera que pyriproxyfen es un controlador químico con efecto residual. Esta información contribuye como precedente a futuras investigaciones y al planteamiento de estrategias eficientes y eficaces en el control focal y/o control de criaderos.
